# Remote Respiratory Monitoring in the Time of COVID-19

**DOI:** 10.3389/fphys.2020.00635

**Published:** 2020-05-29

**Authors:** Carlo Massaroni, Andrea Nicolò, Emiliano Schena, Massimo Sacchetti

**Affiliations:** ^1^Unit of Measurements and Biomedical Instrumentation, Department of Engineering, Università Campus Bio-Medico di Roma, Rome, Italy; ^2^Department of Movement, Human and Health Sciences, University of Rome “Foro Italico”, Rome, Italy

**Keywords:** respiratory rate, remote patient monitoring, technologies, vital signs, accurate measurement, coronavirus

## Introduction

The COVID-19 pandemic has created an unprecedented need for remote patient monitoring. At the time of writing this article, the majority of countries worldwide are on lockdown to minimize the spread of the virus, and most of the patients who tested (or are suspected to be) positive for COVID-19 are in self-isolation at home. Even robust healthcare systems are facing a shortage of healthcare professionals, personal protective equipment, beds, and mechanical ventilators in intensive-care-units (ICU) (Kissler et al., 2020), thus highlighting the need for alternative medical solutions, including remote patient monitoring (Alwashmi, 2020; FDA, 2020; Ohannessian et al., 2020). New policies have therefore been introduced to promote the development of monitoring devices (FDA, 2020), thus creating favorable opportunities to improve the remote monitoring of some overlooked vital signs. This is especially the case for respiratory rate (RR), which is currently poorly recorded despite its relevance in the context of COVID-19.

Current COVID-19 guidelines suggest measuring resting RR to inform triage decisions, diagnosis, prognosis, and as a criterion for ICU admission and for the early recognition of COVID-19 patient deterioration. The World Health Organization indicates that a resting value of RR > 30 breaths/min is a critical sign for the diagnosis of severe pneumonia in adults, while the cut-off value for children varies according to age (World Health Organization, 2020). At triage, RR values are used to support the assignment of patients to different categories and to make decisions on the use of supplemental oxygen (Ayebare et al., 2020; Italian Thoracic Society Italian Respiratory Society, 2020). The treatment of patients affected by acute respiratory insufficiency from COVID-19 is also tailored considering RR values (Italian Thoracic Society Italian Respiratory Society, 2020). Furthermore, RR helps with the timely recognition of COVID-19 patient deterioration, thus contributing to the implementation of early intervention strategies (Sun et al., 2020). Resting RR values also contribute to the prognosis of COVID-19 patients as ICU admission and mortality are associated with significantly higher RR values compared to non-ICU patients and survivors (Huang et al., 2020; Zhou et al., 2020). Besides, the normalization of resting RR is among the signs used to quantify the time to clinical recovery from COVID-19 infection (Al-Tawfiq et al., 2020).

The clinical relevance of RR mandates the improvement of the accuracy of RR measurements in response to the COVID-19 pandemic. RR measurements too often rely on manual counting in the clinical setting and are often poorly performed out of the hospital. For instance, guidelines for telephone consultation of COVID-19 patients recognize the fundamental role of RR in the assessment of patients, but propose unsatisfying solutions for its remote assessment (Greenhalgh et al., 2020). Since patients usually have no direct access to respiratory devices, RR is self-reported by answering the question “Is your breathing faster, slower, or the same as normal?” (Greenhalgh et al., 2020). However, experimental data discourage the self-report of RR and highlight how measurement awareness affects resting RR values (Hill et al., 2018). On the other hand, several sensors and techniques can be used for the accurate remote monitoring of RR; some of these are ready-to-use in the face of the COVID-19 challenge, while others deserve further consideration in the future.

## Technological Solutions for Remote Respiratory Rate Monitoring

**Figure 1:**
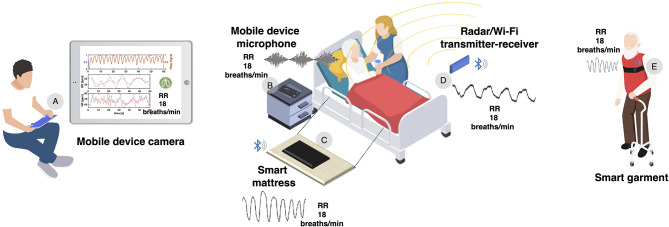
Technological solutions that can be used for the remote RR monitoring of COVID-19 patients. **(A)** The built-in camera of a smart device can be used to record RR from the respiratory-induced chest wall movements or superficial changes in face perfusion of a seated patient. **(B)** The built-in microphone of a smartphone can be used to record RR from the breathing sounds of the patient. **(C)** An instrumented mattress can be adopted for continuous RR monitoring by registering the breathing-related chest wall movements of the patient. **(D)** Radio wave or Wi-Fi signal sources and receivers can be used for registering RR values through the modulation of the transmitted signals by respiratory-related thoracic movements (no body sensors are needed). **(E)** A smart garment (e.g., a strap with conductive strain sensors) can be used to record RR continuously from the respiratory-related periodic changes in chest wall circumference, even during daily activities (e.g., walking).

The abundance of techniques currently available can satisfy the various needs of COVID-19 patients with different severities of symptoms (see Figure 1). We can differentiate techniques for patients that need (1) periodic short-term screening or (2) continuous monitoring. The majority of COVID-19 patients have mild symptoms and can be screened periodically (e.g., twice a day) for vital signs. These patients can take advantage of emerging technologies exploiting built-in cameras of smartphones, tablets, and laptops to record RR from the respiratory-induced chest wall movements or superficial changes in face perfusion (Poh et al., 2011; Brüser et al., 2015). By registering a short video capturing the torso area or the face of the seated patient, RR (and other vital signs like heart rate) can be streamed to healthcare professionals using an internet connection (Brüser et al., 2015). This technique is discreet, available in the market, and shows medical-grade accuracy (error <1 breaths/min) when the patient is stationary (Massaroni et al., 2019a). The wide spread use of smart devices makes camera-based solutions immediately available, relatively low-cost, and user friendly. Furthermore, the availability of smart devices is becoming fundamental in the time of COVID-19, and healthcare systems can benefit from it.

For patients needing continuous monitoring (e.g., older adults, patients with comorbidities or more severe symptoms), prolonged acquisition through camera-based methods is not recommended given the higher amount of data to be processed. In these cases, smart devices offer another ready-to-use technological solution, i.e., the recording of breathing sounds using built-in microphones (Brüser et al., 2015; Nam et al., 2015). Currently available systems have implemented this technology for continuous RR monitoring and unobtrusive sleep apnea detection in quiet environments, with good results in terms of accuracy (error <1%) (Nam et al., 2015). Other solutions available in the market monitor RR continuously with strain or pressure sensors installed underneath mattresses, under bedposts, and on the seating area and backrest of chairs (Watanabe et al., 2005; Brüser et al., 2015). These solutions are discreet, relatively low-cost (~100€), and show medical grade-accuracy (error <2 breaths/min) (Brüser et al., 2015). Furthermore, promising technologies for continuous RR monitoring exploit the modulation of radio waves and Wi-Fi signals by respiratory-related thoracic movements (Lai et al., 2011; Abdelnasser et al., 2015). These waves or signals can be generated by radio sources or traditional modems for internet connection, and have been used to monitor the RR of patients in different postures (seated, standing and lying on the bed) (Brüser et al., 2015). Some of these technological solutions are incredibly small, but are still under development and unavailable in the market (Brüser et al., 2015).

Patients that need continuous vital sign monitoring, even during everyday-life activities, can be equipped with wearable devices like smart garments (e.g., t-shirts or chest bands embedding sensors) (Massaroni et al., 2019b). Unlike most of the aforementioned technologies, smart garments may provide accurate and robust RR values even during daily activities. Several commercial solutions are available in the market with prices around 300€, although further efforts are required to advance the field of respiratory monitoring with wearable devices (Massaroni et al., 2019b). Altogether, a variety of technological solutions are already available for the accurate remote monitoring of RR, and a multidisciplinary approach is required to implement these techniques effectively in medical surveillance programs.

## Discussion

Accurate remote RR monitoring is expected to play an important role in the context of the COVID-19 pandemic. It may facilitate healthcare assistance for self-isolated COVID-19 patients as well as for all patients that have restricted access to medical services in this time of crisis. The improvement of remote patient monitoring would also favor the implementation of timely and cost-effective healthcare services, including the early warning of patient deterioration, remote triage, and home monitoring of COVID-19 patients discharged from hospitals. This would help mitigate the burden on hospitals, decrease the risk of infection for healthcare professionals, and thus reduce virus transmission. The availability of a large number of accurate RR data will also contribute to improving the development of predictive models for the risk of hospital admission, and the development of diagnostic and prognostic models. Finally, we argue that an accurate remote monitoring of RR is essential and should be performed alongside the monitoring of other vital signs. Ready-to-use technological solutions are available to accomplish this goal. Effective RR monitoring would be an important contribution to facing the current COVID-19 crisis and managing similar scenarios that may possibly occur in the next months or years (Kissler et al., 2020).

## Author Contributions

CM, AN, ES, and MS contributed to the conception and design of the work, manuscript writing, critical revision of the article, and approval of the final version of the article.

## Conflict of Interest

The authors declare that the research was conducted in the absence of any commercial or financial relationships that could be construed as a potential conflict of interest.
